# TMS–EEG Co-Registration in Patients with Mild Cognitive Impairment, Alzheimer’s Disease and Other Dementias: A Systematic Review

**DOI:** 10.3390/brainsci11030303

**Published:** 2021-02-27

**Authors:** Raffaele Nardone, Luca Sebastianelli, Viviana Versace, Davide Ferrazzoli, Leopold Saltuari, Eugen Trinka

**Affiliations:** 1 Department of Neurology, Hospital of Merano (SABES-ASDAA), 39012 Merano-Meran, Italy; 2 Department of Neurology, Christian Doppler Klinik, Paracelsus Medical University, 5020 Salzburg, Austria; e.trinka@salk.at; 3 Spinal Cord Injury and Tissue Regeneration Center, 5020 Salzburg, Austria; 4 Karl Landsteiner Institut für Neurorehabilitation und Raumfahrtneurologie, 5020 Salzburg, Austria; 5 Department of Neurorehabilitation, Hospital of Vipiteno (SABES-ASDAA), 39049 Vipiteno-Sterzing, Italy; luca.sebastianelli@sabes.it (L.S.); viviana.versace@sabes.it (V.V.); davide.ferrazzoli@sabes.it (D.F.); leopold.saltuari@gmail.com (L.S.); 6 Research Unit for Neurorehabilitation South Tyrol, 39100 Bolzano, Italy; 7 Centre for Cognitive Neurosciences Salzburg, 5020 Salzburg, Austria; 8 University for Medical Informatics and Health Technology, UMIT, 6060 Hall in Tirol, Tirol, Austria

**Keywords:** transcranial magnetic stimulation, electroencephalography, Alzheimer’s disease, brain connectivity, plasticity

## Abstract

An established method to assess effective brain connectivity is the combined use of transcranial magnetic stimulation with simultaneous electroencephalography (TMS–EEG) because TMS-induced cortical responses propagate to distant anatomically connected brain areas. Alzheimer’s disease (AD) and other dementias are associated with changes in brain networks and connectivity, but the underlying pathophysiology of these processes is poorly defined. We performed here a systematic review of the studies employing TMS–EEG co-registration in patients with dementias. TMS–EEG studies targeting the motor cortex have revealed a significantly reduced TMS-evoked P30 in AD patients in the temporo-parietal cortex ipsilateral to stimulation side as well as in the contralateral fronto-central area, and we have demonstrated a deep rearrangement of the sensorimotor system even in mild AD patients. TMS–EEG studies targeting other cortical areas showed alterations of effective dorsolateral prefrontal cortex connectivity as well as an inverse correlation between prefrontal-to-parietal connectivity and cognitive impairment. Moreover, TMS–EEG analysis showed a selective increase in precuneus neural activity. TMS–EEG co-registrations can also been used to investigate whether different drugs may affect cognitive functions in patients with dementias.

## 1. Introduction

The pathology related to Alzheimer’s disease (AD) leads to cortical large pyramidal neuron degeneration [1] with subsequent impairment of functional connectivity [2].

In particular, amyloid-β (Aβ) plaques and tau-related neurofibrillary tangles have been found to be associated with focal synaptic disruption, which leads not only to regional brain structural changes, but also to abnormal functional connectivity between anatomically distinct brain areas, neuronal circuits and pathways [3,4].

Magnetic resonance imaging (MRI) and positron emission tomography have demonstrated important brain network changes in neurodegenerative diseases, including AD, but the underlying neurophysiological pathways driving pathological processes are still incompletely understood. Several neurophysiological techniques, including electroencephalography (EEG), evoked and event-related potentials (EP/ERP), magnetoencephalography (MEG) and transcranial magnetic stimulation (TMS), can provide accurate information about the normal and abnormal brain aging, thus facilitating non-invasive analysis of cortico–cortical connectivity as well as neuronal synchronization of firing and coherence of rhythmic oscillations at different frequencies. TMS may reveal local excitability changes in cortical and transcortical motor circuitries, while EEG and MEG are able to reveal cortical neural synchronization and connectivity with excellent temporal and spatial resolution. These novel approaches can help in detecting network disruption in neurodegenerative disease.

TMS also allows the quantification of motor system excitability and can create transient “virtual” functional lesions of networks subserving various cognitive functions such as attention and memory.

Integration of TMS with EEG [5,6,7] may offer the possibility of providing real-time information on cortical reactivity and distributed network dynamics through the analysis of TMS-evoked potentials. Indeed, TMS–EEG co-registration enables us to explore the interactions between distinct neural areas during cognitive processes, the causal role of specific brain area in behaviour, as well as the connectivity and relationship between activities in distinct brain regions. By means of combined TMS–EEG, the causal role can be established in the interactions between cortical areas and neuronal networks.

With this integrative approach, from the stimulated area action potentials can propagate to the interconnected brain areas directly or indirectly, thus allowing for the evaluation of causal correlation, temporal evolution of interactions between and within different brain regions and the excitatory or inhibitory nature of these connections.

In particular, the combination of TMS with EEG may offer a direct non-invasive method to explore cortical reactivity and functional connectivity in physiological and pathological conditions [8,9,10,11,12,13,14] because the TMS-evoked cortical response extends to anatomically distant but connected areas. This neurophysiological approach for direct recording of EEG responses to TMS of a given scalp site with millisecond resolution allows one to study non-invasively and simultaneously cortical excitability and time-solved effective connectivity at the cortical level and within the same time-window [15]. This is not affected by different confounding effects, such as attentional/cognitive individual bias or the functional state of peripheral strictures (in particular the spinal cord or the neuromuscular unit). In fact, a network of neuronal connections is engaged when TMS-induced activation propagates from a stimulation site to other brain regions, and the summation of synaptic potentials generates deflections of alternating polarity in scalp EEG signals. The signals begin at a few milliseconds and last about 300 ms after TMS, at first in the form of rapid oscillations and afterwards as lower-frequency waves [15]. The amplitude, latency and scalp topography of single pulse TMS-evoked EEG responses after M1 stimulation have been clearly illustrated in healthy subjects [12,14,16,17,18,19] and are thought to be related to the stimulation intensity and functional state of the targeted cortex [12,20]. Therefore, they reflect directly and timely the excitability and connectivity properties of the stimulated cortex [21].

In particular, integrated approaches employing TMS together with other neurophysiological methods, such as high-density EEG, structural and functional imaging as well as the identification of biological markers, are promising tool for non-invasive, large-scale and low-cost assessment of at-risk populations [22,23,24,25].

We aimed to review here the TMS–EEG co-registration studies in patients with mild cognitive impairment (MCI), AD and other dementias.

## 2. Methods

The MEDLINE, accessed by Pubmed (1966–November 2020) and EMBASE (1980–November 2020) electronic databases were searched using the medical subject headings (MeSH) and free terms: “dementia”, “mild cognitive impairment”, “Alzheimer’s disease”, “transcranial magnetic stimulation”, “repetitive transcranial magnetic stimulation”, “electroencephalography”, “magnetoelectroencephalography”, “event-related potential/s”, “evoked potential/s”, “co-registration”.

Only original articles were considered eligible for inclusion, while single case reports and review articles were excluded. The search was limited to studies written in English. The reference lists of the retrieved full-text articles were searched for additional publications. In the case of missing or incomplete data, principal investigators of included trials were contacted and additional information requested.

The titles and abstracts of the initially identified studies were screened by two reviewers with experience in systematic review protocols involved in designing the literature search strategy to determine whether they satisfied the selection criteria.

To evaluate the quality of the manuscripts, a thorough evaluation has objectively judged all their aspects, including objective and hypothesis, scientific background and explanation of rationale, eligibility criteria for participants, sample size, dates defining the periods of recruitment and follow-up, baseline demographic and clinical characteristics of each group, statistical methods used to compare groups, precise details of the interventions and how and when they were actually administered and clearly defined primary and secondary outcome measures. The methodological quality of each study as well as risk of bias, focusing on blinding, were evaluated. The Risk of bias was assessed using the Cochrane risk of bias tool [26].

This search strategy yielded 10 articles.

The demographic characteristics of the patients and principal findings of the selected studies are summarized in Table 1.

**Figure 1:**
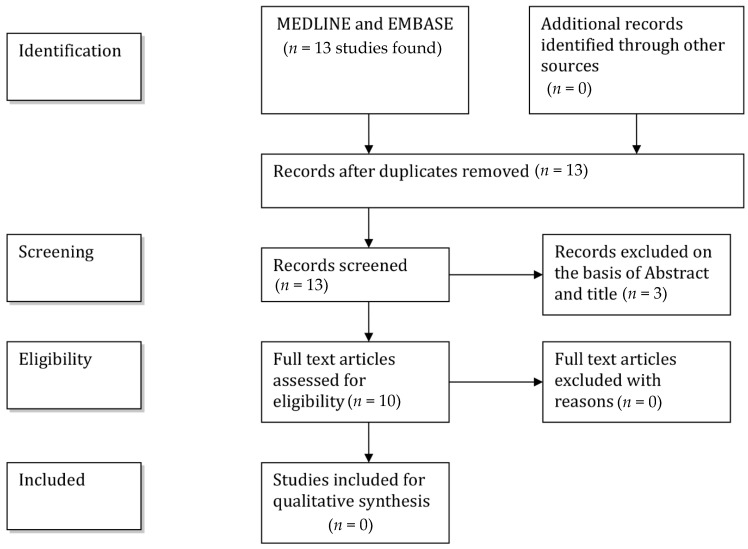
Flowchart showing the selection/inclusion process.

A flow-chart (Figure 1) illustrates the selection/inclusion process.

## 3. TMS–EEG Co-Registration Studies

### 3.1. TMS–EEG Studies Targeting the Motor Cortex

Julkunen and colleagues first assessed the usefulness of navigated TMS-evoked EEG responses in exploring neuronal activity as well as cortical connectivity in MCI and AD [27]. Five patients with MCI, five patients with AD and four healthy control subjects were examined. Fifty TMS-pulses at a stimulus intensity of 110% of the resting motor threshold (RMT) were applied to the hand area of primary motor cortex (M1) with navigated TMS. Spreading of navigated TMS-evoked neuronal activity was monitored with a compatible 60-channel EEG and analysed in time, frequency and spatial domains.

The P30 is a well distinguishable, TMS-evoked response in EEG, which has been suggested to involve pathways between subcortical structures such as the thalamic nuclei or basal ganglia and cortex [36] by “real” cortical TMS-induced potentials [17].

**Figure 2:**
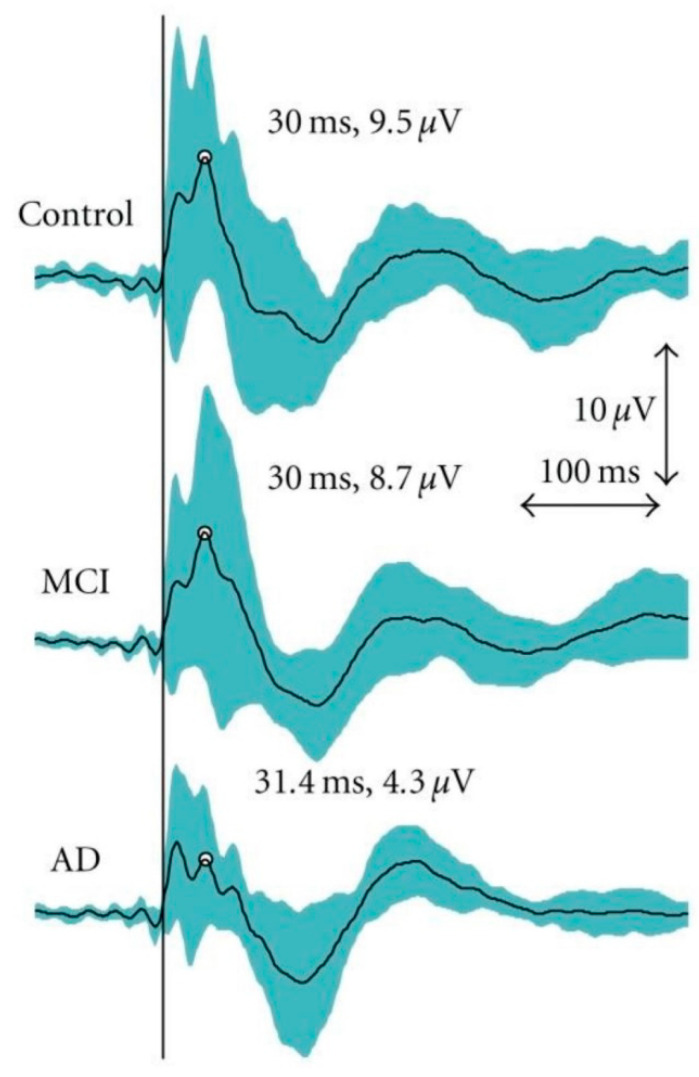
Grand average curves for transcranial magnetic stimulation (TMS)-evoked electroencephalography (EEG) responses as measured from the central electrode (CZ). The mean peak for the P30 has been indicated. However, P30 was analysed for individuals from the electrode chosen based on the shortest latency and clearest identification on the stimulated hemisphere. The turquoise area represents the 95% confidence interval for the TMS–EEG responses. The vertical black line indicates the moment of stimulation. Reproduced with permission from Julkunen et al., 2011 [28].

The authors found in the AD patients a significantly decreased TMS-evoked P30 (time-locked response 30 ms after the TMS) in the temporo-parietal cortex ipsilateral to stimulation side and in the contralateral fronto-central area corresponding to the sensorimotor network (Figure 2), which is anatomically interconnected with the stimulated M1, as well as a significantly reduced amplitude of the N100 in the MCI patients in comparison to the healthy controls. Therefore, the combination of TMS and EEG showed relevant abnormalities in functional cortical excitability and connectivity in the AD subjects. This pilot study suggests that TMS–EEG co-registration may provide a promising tool for evaluating the degree and progression of dementia.

In a subsequent study, the same authors re-analysed the small sample data from their first study with the aim to assess the sensitivity of the TMS–EEG features to distinguish healthy subjects from MCI and AD. Additionally, the correlation between the TMS–EEG response characteristics and the scores of the dementia rating scales were considered to assess the severity of cognitive decline in these subjects. The TMS–EEG response P30 amplitude correlated with cognitive impairment and exhibited good specificity and sensitivity in identifying control subjects from those with MCI or AD. However, because of the small sample size, further studies are required to confirm these initial findings [28].

EEG and TMS were simultaneously studied in order to assess the frontal cortex plasticity and connectivity changes during healthy and pathological aging [29]. Consequently, the TMS-induced EEG potentials recorded in healthy elderly subjects were compared with the ones collected in healthy young subjects and in AD patients. The EEG response to TMS of the left superior frontal cortex is not affected by physiological aging but is remarkably altered in subjects with cognitive decline.

**Figure 3:**
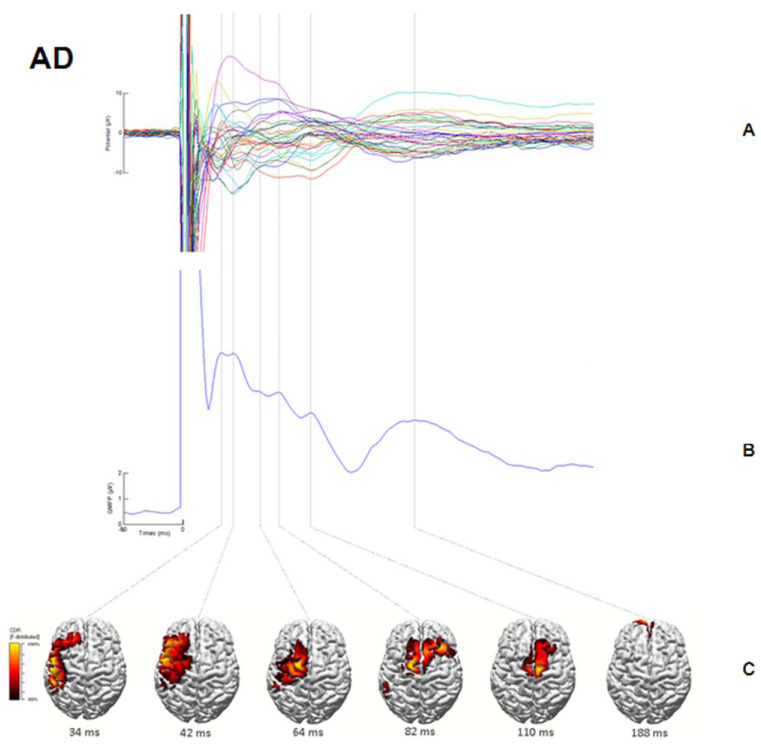
Alzheimer’s disease (AD) patients. (**A**) Averaged TMS-evoked potentials recorded at all electrodes, superimposed in a butterfly diagram. (**B**) Global activation induced by TMS as evaluated by the global mean field power (GMFP). (**C**) Source localization of the activity occurring during each peak in the GMFP calculated using standardized, low-resolution brain electromagnetic tomography (sLORETA) and plotted on the cortical surface. At each time point, the results were auto-scaled and thresholded at 80% to highlight maximum current sources. Reproduced with permission from Ferreri et al., 2016 [30].

In another TMS–EEG co-registration study, Ferreri et al. demonstrated an increased excitability in the sensorimotor cortex of mild AD, despite clinically evident motor manifestations were lacking [30]. This finding may be related to a stronger response to stimulation in a specific time window, possibly determined by locally acting reinforcing circuits, while network activity and connectivity are decreased (Figure 3). These changes can be interpreted as a compensatory mechanism enabling the preservation of sensorimotor programming and execution over a long period of time, independently of the disease progression.

### 3.2. TMS–EEG Studies Targeting Other Cortical Areas

Gandelman-Marton et al. evaluated long-term electrophysiological effects of repetitive TMS (rTMS) interlaced with cognitive training (COG) on quantitative EEG, before and after each treatment phase in seven subjects with mild AD [31]. If delivered repetitively, TMS can influence cortical excitability and neuronal activity [37,38,39]. After 54 sessions (4.5 months) of treatment, a significant enhancement of delta activity over the temporal region was detected in comparison with pre-treatment values. Non-significant changes of the log EEG power were observed for alpha band over the frontal and temporal areas; beta band over the frontal region; theta band over the frontal, temporal and parieto-occipital regions; and delta band over the frontal and parieto-occipital areas. Conversely, EEG power decreases were observed for alpha over the parieto-occipital regions and for beta over the temporal as well as parieto-occipital regions, but the differences were also not significant.

Log alpha power over the frontal and temporal areas at 6 weeks correlated positively with Mini-Mental State Examination (MMSE) scores at 6 weeks and 4.5 months, and log alpha power over the parieto-occipital regions with MMSE scores at 6 weeks. There was a negative correlation between log alpha power over the frontal and temporal regions at 6 weeks and baseline Alzheimer’s Disease assessment Scale–Cognitive Subscale scores. rTMS combined with cognitive training exerts long-term effects on quantitative EEG in subjects with mild AD.

**Figure 4:**
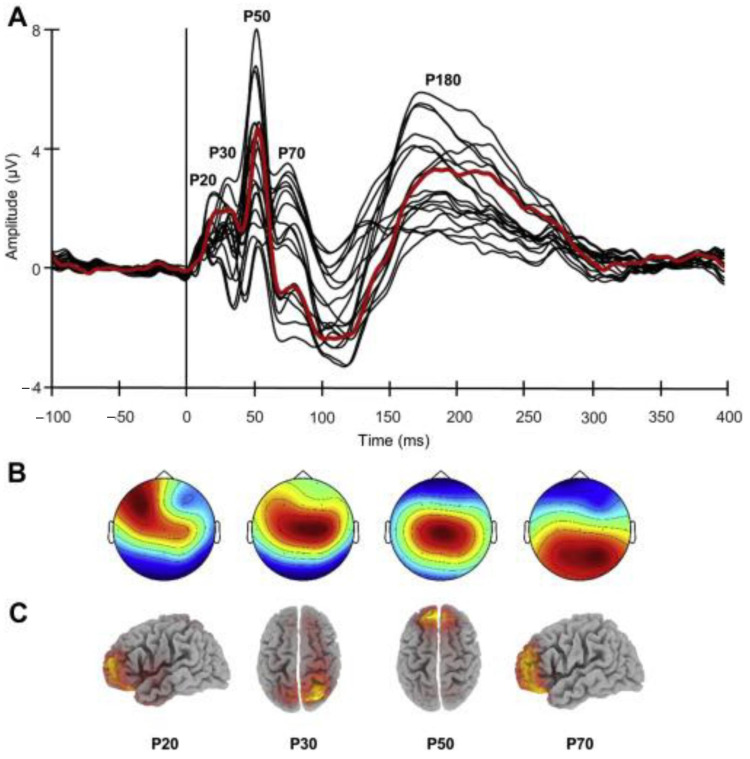
TMS–EEG results. (**A**) Butterfly plot of the grand-averaged TMS-evoked potentials (TEP) from all the electrodes showing the main components elicited by left dorsolateral prefrontal cortex stimulation. The signal from the target site of stimulation (electrode F3) is depicted by the red line. (**B**) Topographic distribution. (**C**) sLORETA source localization for each TEP component before 100 ms. (For interpretation of the references to color in this figure legend, the reader is referred to the Web version of this article). Reproduced with permission from Bagattini et al., 2019 [34].

The correlation between dorsolateral prefrontal cortex (DLPFC) connectivity (derived from TMS–EEG co-registration) and cognitive impairment, as evaluated with MMSE and a face–name association memory task, has been explored in 26 patients with AD [34]. The amplitude of TMS–EEG evoked component P30, which originates in the right superior parietal cortex, predicted MMSE and face–name memory scores. Indeed, higher P30 amplitudes correlated with lower cognitive performances (Figure 4). These results suggest that advancing disease severity might be associated with effective connectivity enhancement involving long-distance fronto-parietal connections and may reflect maladaptive pathogenic mechanisms that arise from a damaged balance between the excitatory and inhibitory activity in anterior and posterior regions.

**Figure 5:**
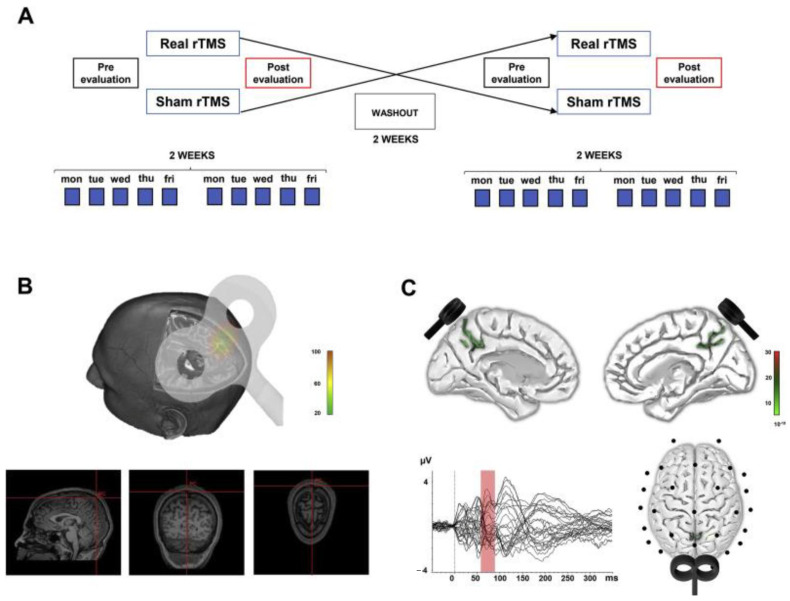
Experimental design and source analysis reconstruction. (**A**) AD patients (*n* = 14) were randomly assigned to either a first neuronavigated rTMS or control stimulation (sham) arm. rTMS/sham was delivered daily in a 10-session course, Monday to Friday, for a total duration of two weeks. A two-week washout interval was then applied, following which patients were crossed over to the alternative study arm for two additional weeks. The order of assignment to either study arm was counterbalanced across patients. (**B**) rTMS was applied over the PC at 20 Hz (1600 stimuli per day), using a neuronavigation system to ensure that the same spot was constantly stimulated across sessions. (**C**) Source analysis of TMS-evoked EEG activity revealed a bilateral activation of the PC, as reconstructed at the peak fit of cortical response between 60 and 90 ms from single-pulse TMS over PC. Reproduced with permission from Koch et al., 2018 [33].

Using a multimodal approach, the effects of high-frequency rTMS of the PC on cognitive functions, as evaluated by the Alzheimer’s Disease Cooperative Study Preclinical Alzheimer’s Cognitive Composite, have been examined in a two-week, randomized, sham-controlled, double-blinded trial in 14 patients with early AD [40]. Furthermore, TMS–EEG co-registration was used to detect changes in brain connectivity. rTMS applied over PC lead to a significant improvement in episodic memory, but not in other cognitive domains. Analysis of TMS–EEG signal in patients’ PC showed an increase in neural activity and of brain oscillations in the beta band as well as changes in the functional interactions between the PC and medial frontal areas within the default mode network (DMN) (Figure 5). These findings indicate that high-frequency rTMS of the PC may represent a promising, non-invasive treatment for memory dysfunction in subjects at early stages of AD. This clinical improvement is accompanied by a modulation of brain connectivity, consistent with the pathophysiological model of brain disconnection in AD.

Kumar et al. aimed at determining whether patients with AD present impairments of DLPFC plasticity [32], measured as potentiation of cortical-evoked activity using paired associative stimulation, which consists in repeated pairs of electrical stimulation of a peripheral nerve (commonly the median nerve) followed by TMS delivered over the contralateral M1 hand area [41]. The authors also compared working memory between patients with AD and controls in order to determine whether DLPFC cortical-evoked activity was associated with working memory. This study demonstrated impaired DLPFC plasticity in subjects with AD. These findings suggest that DLPFC plasticity can be used as an index of DLPFC function and a potential treatment target to increase DLPFC function and working memory in subjects with AD.

In these latter studies using rTMS and reporting the improvement of episodic memory, the effect can be was solely attributed to rTMS, since the participants were taking no drugs [40], or were in treatment with a stable dose of a cognitive enhancer [32].

### 3.3. Evaluation of Drugs Effects by Means of TMS–EEG Co-Registration

Since an abnormal dopaminergic transmission is thought to contribute to cognitive impairment in AD, Koch and colleagues investigated whether treatment with dopaminergic agonists may affect cognition in these subjects [33]. In a monocentric, randomized, double-blind, placebo-controlled study, patients with mild to moderate AD received a rotigotine 2 mg transdermal patch for 1 week followed by a 4 mg patch for 23 weeks (*n* = 47) or a placebo transdermal patch for 24 weeks (*n* = 47). Among 94 randomized patients randomized, 78 (83%) completed the study. Rotigotine, as compared with placebo, exerted no significant effect on the primary end-point. Indeed, estimated mean change in Alzheimer’s Disease Assessment Scale–Cognitive Subscale score was 2.92 (95% CI, 2.51–3.33) for the rotigotine group and 2.66 (95% CI, 2.31–3.01) for the placebo group. For the secondary outcomes, significant estimated mean changes between groups for the Alzheimer’s Disease Cooperative Study–Activities of Daily Living score (−3.32 (95% CI, −34.02 to −32.62) for rotigotine and 7.24 (95% CI, −37.84 to −36.64) for placebo) and FAB score (0.48 (95% CI, 0.31 to 0.65) for rotigotine and −0.66 (95% CI, −30.80 to −30.52) for placebo) were observed. No longitudinal change in NPI scores (1.64 (95% CI, 1.06–2.22) for rotigotine and 1.26 (95% CI, 0.77–1.75) for placebo group) was found.

The analysis of EEG recordings revealed that prefrontal cortical activity was enhanced in rotigotine but not in the placebo group. Additionally, adverse events were more frequent in the rotigotine group.

Since a link has been postulated between neuroinflammation and frontotemporal dementia (FTD), a neurodegenerative disease for which effective pharmacological treatment is lacking, Assogna et al. aimed to assess the effects of palmitoylethanolamide (PEA) combined with luteoline (PEA–LUT), an endocannabinoid with anti-inflammatory and neuroprotective properties, on behaviour, cognitive abilities and cortical activity in patients affected by FTD [35]. In seventeen subjects with a diagnosis of probable FTD, neuropsychological and neurophysiological evaluations were carried out at baseline and after 4 weeks of PEA–LUT 700 mg × 2/day. Effects on cognitive function were assessed by MMSE, NPI, FAB, Screening for Aphasia in Neurodegeneration, Activities of Daily Living–Instrumental Activities of Daily Living and Frontotemporal Lobar Degeneration–modified Clinical Dementia Rating scale. To investigate in vivo neurophysiological effects of PEA–LUT, the authors used paired-pulse TMS and rTMS protocols evaluating long-term potentiation (LTP)-like cortical plasticity, short-interval intracortical inhibition, long-interval intracortical inhibition (LICI) and short-latency afferent inhibition. Moreover, TMS in combination with EEG was used to assess the effects on frontal lobe cortical oscillatory activity. After treatment with PEA–LUT, an improvement in NPI and FAB scores was reported. Neurophysiological examination revealed a reversal of the LICI abnormalities, especially at interstimulus interval (ISI) of 100 ms, indicating a modulation of GABA_B_ activity. TMS–EEG co-registration showed a marked enhancement of TMS-evoked frontal lobe activity and of high-frequency oscillations in the beta/gamma range. Therefore, PEA–LUT could decrease behavioural disturbances and improve frontal lobe functions in FTD patients through by modulating the cortical oscillatory activity and GABA_B_ergic transmission.

## 4. Discussion

The results of these reviewed articles are consistent with those of some neuroimaging and electrophysiological studies reporting that in AD the clinical manifestations are attributable not only to a regional gray matter degeneration, but also with a disruption in the interactions between brain areas due to compromised large-scale connections, thus supporting the notion that AD can be considered a “disconnection syndrome” [42].

TMS-evoked response at around 30–50 ms was significantly reduced over widespread brain regions in AD patients. This finding likely reflects a dysfunction of a large-scale sensorimotor networks [27]. All the basic components in the TMS-evoked EEG response were detected in AD, MCI and control groups, but the early responses, which likely reflect cortical excitability and functional connectivity, were reduced only in AD patients. The TMS-induced P30 peak [16,43] was lower in the AD group in comparison to the control and MCI groups. Besides of the reduced reactivity and/or connectivity between brain areas, the decreased responses may also indicate an impaired synchronization of the EEG activity in the AD group than in the healthy or MCI subjects. This finding is consistent with a previous study revealing that decreased EEG synchronization was related to cognitive decline [44]. The magnitude of TMS-evoked EEG responses is presumed to indicate principally the synchronized activation of underlying neuronal population and therefore the functional connectivity of the neuronal pathways arising from M1. Highest peak amplitudes (P30, N100 and P200) have also been observed in some other studies [16,36,43,45] and are thought to provide an index of functional connectivity from the site of TMS to the sources of the evoked responses.

Casarotto et al. clearly demonstrated that TMS evoked potential are not affected by physiological aging by itself, unless a cognitive impairment is associated [29]. Indeed, only in AD patients a decreased global EEG response to TMS between 30 and 50 ms, as well as a reduced current density in the stimulated cortex approximately 30 ms after TMS, were found.

Navigated TMS–EEG may thus be helpful in identifying and tracking abnormal changes in the state of cortical circuitries across the lifespan.

By using TMS–EEG co-registration, Ferreri et al. have also demonstrated a deep rearrangement in the sensorimotor system of mild AD patients without who do not exhibit motor symptoms [30].

A marked increase in cortical excitability in these patients possibly suggests a reorganization of cortical plasticity and connectivity with the recruitment of additional neural sources, as well as the activation of reverberant local circuits and their integration in the distributed network underlying sensorimotor functions. This plastic rearrangement is enabled by the peculiar organization of the sensorimotor system consisting of a distributed network with a replicated topographic organization of the same body part [46]. These changes could reflect a compensatory mechanism in the attempt to preserve the sensorimotor programming and execution over a long period of time in spite of disease progression of the degenerative process. Such a plastic reorganization can play the same role in largely preserving the sensorimotor functions in mild AD patients, but this should be clarified in further studies.

Anyway, together with a deep understanding of the above mentioned compensatory mechanisms, the emerging possibility to influence cortical excitability by means of non-invasive brain stimulation techniques in healthy and pathological brains [47,48,49] could open new and promising therapeutic perspectives in neurodegenerative disorders that present with dementia.

Previous findings from TMS–EEG co-registration studies targeting the M1 in subjects with AD, even if partly conflicting, have underscored significant abnormalities in the P30, an early positive EEG response which is localized by the sLORETA algorithm to the right superior parietal lobule [27,28].

Gandelman-Marton and co-workers first evaluated the electrophysiological effects of multiple rTMS-COG sessions in subjects with mild AD [29]. Analysis of the EEG background activity revealed significant enhancement of delta activity over the temporal region after 54 sessions of treatment compared with pre-treatment values. Additionally, alpha power particularly over the frontal and temporal regions at 6 weeks correlated significantly with the neuropsychological evaluations at 6 weeks and 4.5 months.

The EEG reflects the synaptic activity of large cortical pyramidal neurons, triggered by cortical and subcortical inputs [50]. Slow-frequency, large-voltage EEG rhythms in the delta range can be determined by intrinsic deactivated cortical networks, while the appearance of fast-frequency, low voltage rhythms is thought to depend on subcortical input [51]. The cholinergic fibers that originate in the basal forebrain and target the cerebral cortex [52], as well as the hippocampus and the thalamic reticular nucleus [53], can induce a suppression of slow-frequency and large-voltage EEG rhythms, which are replaced by fast-frequency and low-voltage rhythms. The EEG abnormalities reflect the progressive structural changes in the cortex of AD patients and are associated with the cognitive decline [54].

Bagattini and colleagues investigated the correlation between prefrontal TMS-evoked activity and disease severity in subjects with mild and moderate AD patients. They reported that the P30 amplitude is enhanced in patients with more severe cognitive decline (lower MMSE score) and that this augmentation can predict disease severity. This finding indicates an inverse association between prefrontal-to-parietal connectivity and cognitive impairment; indeed, left DLPFC connectivity becomes stronger with progress of disease severity advances. This finding is corroborated by functional MRI (fMRI) studies that demonstrated enhanced connectivity of prefrontal areas in AD [55,56]. Zhang et al. explored functional connectivity between core regions underlying episodic memory retrieval and found that the strength of connections between left frontal and right parietal areas was associated with worsen disease severity [56]. Notably, the AD-associated abnormalities of the left DLPFC effective connectivity seem to be selective. P50 and P70, which are generated in other areas of the prefrontal cortex, exhibited no significant correlation with AD severity. The left DLPFC is involved in several networks such as fronto-parietal connections and DMN; it has been also demonstrated that TMS over DLPFC can activate both networks [57]. The selective association with the P30, without other effects on the evoked responses within the 100 ms after stimulation, may suggest that only specific networks play a relevant role in the cognitive functions impairment associated with different illness stages. The findings of this study do not allow the identification of the network which is primarily involved in the worsening of AD symptoms, but they paved the way for further research. Cutting-edge TMS–EEG technical improvements could make it possible to tailor more precisely and individually the stimulation site based on patients’ brain scans, and therefore to identify the target network.

High-frequency rTMS, which is thought to increase excitability in the stimulated cortical region, applied over PC can induce a selective improvement in episodic memory in AD patients, thus suggesting the induction of LTP-like cortical plasticity within the PC. In agreement with this hypothesis, TMS–EEG analysis showed a significant enhancement of PC neural activity. The increased activity was evident not only at the site of stimulation but also at a distributed network level. In fact, abnormalities in neural activity were located over two distinct clusters of electrodes: one at local level (PC) and one corresponding to the medial frontal cortex, indicating that rTMS can exert important modulatory effects over a medial parieto-frontal circuit. It should be noted that the topography of this EEG network resembles the anatomical distribution of the DMN, as detected by fMRI [58,59]. Moreover, rTMS provoked significant increases in TMS-induced beta activity, both in terms of power and phase synchronization, which is focused over the medial parietal electrodes underlying the site of stimulation. Since rTMS was delivered at 20 Hz, a frequency that falls within the range of beta oscillations, these findings can be explained by a possible long-lasting entrainment of beta-rhythm provoked by rTMS [13]. Therefore, these results are consistent with models proposing oscillatory activity in the beta range as an efficient cortical frequency, which surveys relevant process information in different brain networks, thus playing a critical role on several memory processes [60].

In rodents, acetylcholinesterase inhibitors (AChE-I) and serotonergic drugs can reverse an abnormal pattern of EEG synchronization. Moreover, the feasibility and reproducibility of TMS challenge have been reported, but information on EEG modulation after TMS is rather limited in these experimental animals [51].

In a randomized clinical trial, the treatment with the dopaminergic agonist rotigotine did not significantly affect global cognitive performances in patients with mild to moderate AD, while the cognitive functions associated with the frontal lobe and the activities of daily living significantly improved. These results indicate that rotigotine treatment may reduce symptoms associated with frontal lobe cognitive dysfunction, thus delaying the impairment of daily living activities.

Rotigotine induced a marked increase in prefrontal cortex activity, as revealed by TMS–EEG recordings [33]. Treatment with rotigotine was also found to enhance the evoked EEG response to TMS, resulting in enhanced oscillatory activity in the range of alfa and beta frequencies.

Multimodal methods should be introduced in order to validate these techniques. Standardization and harmonization of user-friendly acquisition and analysis protocols in larger cohort populations are also required in order to include electrophysiological characteristics as a part of the clinical criteria of AD.

Further studies on the quantitative EEG long-term effects of TMS combined with cognitive training are required to confirm these preliminary findings.

TMS–EEG results in patients with FTD revealed that PEA-LUT treatment leads to significant enhanced cortical activity in left DLPFC and especially in later components of the TMS-induced EP that are likely related to the GABA_B_ inhibitory transmission induced by TMS.

These studies have several limitations. Some of them (i.e., [31]) have been conducted in a relatively small sample, and therefore the results should be replicated in larger cohorts of patients to verify if TMS–EEG can represent a reliable diagnostic biomarker of AD severity. Moreover, longitudinal studies examining long-term changes are required to take a step toward the development and validation of P30.

However, despite these limitations the quality of the reviewed manuscripts was high and the risk of bias overall low.

With the aim of selecting a homogeneous cohort, in the study of Koch et al. cerebrospinal fluid biomarkers were used to support the clinical diagnosis of AD in all participants, in agreement with the current diagnostic criteria [61]. Furthermore, the low number of electrodes utilized for EEG recordings limits the spatial resolution of their conclusions, in particular for the source analysis.

The EEG activity probably provides a more sensitive measure for assessing TMS impact on brain function than behavioural effects. However, a larger sample could be necessary to detect low-magnitude effects on the EEG, even if significant results have been reported in similar TMS-quantitative EEG studies that have used sample sizes of this magnitude [7]. Since the sample size needed to reach 0.8, power was calculated to be 200 patients; a multicenter study is needed to meet sample size requirements. Additionally, some patients received baseline treatment with AchE-I, alone or in combination with an NMDA antagonist; therefore, it is conceivable that changes in the EEG power or in the scores of the neuropsychological tests could depend on medical treatment and not necessarily on TMS-COG.

Contrary to the conventional protocols of combined TMS–EEG studies, in the study of Gandelman-Marton et al. the EEG power was measured at least 24 h after the last TMS-COG session and not within an hour post-stimulation [31]. An extended stimulation EEG recording interval may help in detecting possible long-term EEG effects, and similar timing of EEG recording has been used in pharmaco-EEG studies in patients with AD.

rTMS investigations are particularly suitable in association with scalp recordings of the EEG as a safety measure in monitoring ongoing EEG activity and as a neurophysiologic method in evaluating the specific effects produced by TMS on the EEG or evoked potentials. Interestingly, rTMS can reverse the Aβ1-42-induced abnormalities in gamma oscillation during working memory in adult rats [62].

There is certainly a great need for biomarkers that could reflect, within a very short time period, functional brain dynamic changes, thus providing important information about cognitive impairments.

In conclusion, the above described electrophysiological techniques have the highest time resolution for reflecting brain dynamics in cognitive decline. In particular, the characteristics of TMS–EEG recordings provide a fascinating tool to explore the causal relationships in the interactions between brain areas and can disclose the activation of these connections at the time of stimulation. Therefore, the combination of TMS with EEG (or fMRI) enables clinicians and researchers to directly explore, in vivo in humans, local and network cortical plasticity, as well as to characterize their changes across the age-span in health and during disease progression.

TMS reversibly interfered with higher brain functions during EEG recordings, but few studies have investigated the relationship between the cognitive and EEG effects of TMS.

Since Koch and colleagues demonstrated a reversal of memory impairment following dopamine agonist treatment [40], the combined TMS–EEG analysis could also be applied in patients with Parkinson’s disease. Therefore, this technique can be extended to several neurodegenerative disorders related to memory impairment, and also to evaluate the effects of the therapeutic approaches with non-invasive brain stimulation methods in these disorders.

Non-invasive neuromodulation techniques such as TMS, rTMS and tDCS, combined with other research methods (such as the TMS–EEG co-registration), can thus be used to understand if cortical plasticity can be induced and manipulated by means of non-invasive brain stimulation in healthy adult brains to clarify the relation between induced synaptic plasticity and cognitive plasticity, and how the latter can be sustained by the activity of a “functional neuronal network”. Moreover, it should be of interest to explore the relationships between this network and the condition of the single subject.

## Figures and Tables

**Table 1:** Demographic characteristics of the patients and principal findings of the studies.

Studies	Nr Disease	Age (y)	Gender F/M	Education (y)	DD	MSE	Principal Neurophysiological Findings
Jalkunen et al., 2008 [27]	5 MCI	74.0 ± 8.1	2/3	7.6 ± 3.0	?	25.4 ± 3.2	Significantly reduced P30 in the ipsilateral temporo-parietal area
	5 AD	73.26 ± 8.1	2/3	9.6 ± 2.1		22.0 ± 5.1	and in the contralateral centro-frontal cortex
Jalkunen et al., 2011 [28]	5 MCI	74 ± 8	2/3	7.6 ± 3.0	?	?	P30 amplitude correlated with cognitive decline and showed good specificity
	5 AD	73 ± 8		9.6 ± 2.1	?	?	and sensitivity in identifying subjects with MCI or AD
Casarotto et al., 2011 [29]	9 AD	72 ± 7.1	5/4	10 ± 3.5	?	18 ± 4.5	The EEG response to TMS of the left superior frontal cortex is not
							affected by physiological aging but is altered by cognitive impairment
Ferreri et al., 2016 [30]	12 AD	72.4 ± 5.9	7/5	8 ± 3.5	?	20.8 ± 2.7	The sensorimotor system is hyperexcitable in patients with AD
Gandelman-Marton et al., 2017 [31]	7 AD	75.5 ± 4.3	1/7	11.6 ± 2.7	2.8 ± 1	?	After rTMS significant increase in delta activity over temporal region
							Positive correlation between alfa power and MMSE
Kumar et al., 2017 [32]	32 AD	76.3 ± 6.3	17/15	13.5 ± 3.8	?	22.6 ± 3.2	DLPFC plasticity in patients with AD
Koch et al. 2018 [33]	14 AD	70.0 ± 5.1	7/7	7.2 ± 3.0	?	26.1 ± 1.8	rTMS increased neural activity in PC, enhanced brain oscillations in the beta
							band and modified functional connections between PC and frontal areas
Bagattini et al., 2019 [34]	26 AD	76.5 ± 4.7	23/3	7.7 ± 4.1	?	20.8 ± 2.5	Higher P30 amplitudes predicted poorer cognitive performances
Koch et al., 2020 [33]	94 AD	73.9 ± 5.6	58/36	^1^ 8.5 ± 4.2	?	^1^ 22.9 ± 2,3	Rotigotine increases frontal cortical activity, but did not significantly affect
				^2^ 9.4 ± 4.3		^2^ 23.6 ± 2.4	global cognition
Assogna et al., 2020 [35]	17 AD	62.35 ± 9.4	11/6	12.47 ± 3.41	2.6 ± 1.3	?	Treatment with PEA-LUT restores LICI at ISI 100 ms, increases TMS-evoked
							frontal lobe activity and high-frequency oscillations in beta/gamma range

Nr = number; F = female; M = male; DD = disease duration; MMSE = Mini-Mental State Examination; MCI = mild cognitive impairment; AD = Alzheimer’s disease; DLPFC = dorsolateral prefrontal cortex; PC = precuneus; PEA-LUT = palmitoylethanolamide combined with luteoline; LICI = long-interval intracortical inhibition; ISI = interstimulus interval; ms = milliseconds; ^1^ rotigotina group; ^2^ control group.; ? = not reported.
